# Influence of maximum MLC leaf speed on the quality of volumetric modulated arc therapy plans

**DOI:** 10.1002/acm2.13020

**Published:** 2020-10-12

**Authors:** Jiayun Chen, Weijie Cui, Qi Fu, Haojia Zhang, Xiaodong Huang, Fei Han, Wenlong Xia, Bin Liang, Jianrong Dai

**Affiliations:** ^1^ National Cancer Center/National Clinical Research Center for Cancer/Cancer Hospital Chinese Academy of Medical Sciences and Peking Union Medical College Beijing China; ^2^ Department of Oncology The Affiliated Hospital of Guizhou Medical University Guiyang China

**Keywords:** maximum MLC speed, plan quality, VMAT

## Abstract

**Purpose:**

Maximum leaf speed is a configurable parameter of MLC in a treatment‐planning system. This study investigated the influence of MLC on the quality of VMAT plans.

**Methods:**

Seven MLCs with different maximum leaf speeds (1.0, 1.5, 2.25, 3.5, 5.0, 7.5, and 10 cm/s) were configured for an accelerator in treatment‐planning system. Correspondingly, seven treatment plans, with the identical initial optimization parameter, were designed with the mdaccAutoPlan system. Six nasopharyngeal carcinoma (NPC) patients and nine rectal cancer patients were selected, representing complex and simple clinical circumstances. VMAT plan quality was evaluated with PlanIQ^TM^ software. The results were statistically analyzed with a one‐way analysis of variance (ANOVA) and pairwise comparison tests.

**Results:**

The relative changes of plan scores achieved by the seven configured accelerators, with specific maximum MLC leaf speed (MMLS) for each patient, were studied. Two apparent trends of MMLS influence on VMAT plan scores were observed: Plan scores increased with MMLS; Plan scores increased rapidly when MMLS increased from 1 to 3.5, thus the relative change of plan score decreased in this MMLS range. The stationary point of maximum MLC speed (MMSSP) is defined, for the specific MMLS when the relative changes of plan scores is first <5%, as MMLS increases from 1.0 to 10. For rectal plans, MMSSPs were 2.25 for six patients and 3.5 for the other three patients. For NPC plans, MMSSPs were 3.5 for five patients and 2.25 for one patient.

**Conclusion:**

This work indicates that MMLS directly influences VMAT plan quality in NPC cases and rectal cancer cases. VMAT plan quality improved conspicuously as MMLS increased from 1 to 3.5, VMAT plan quality with marginal improvement when MMLS is above 3.5.

## 1 INTRODUCTION

Volumetric modulated arc therapy (VMAT), first introduced in 2007, was described as a novel technique in radiotherapy.^1^, ^2^ Volumetric modulated arc therapy delivers a highly conformal prescription dose to the target volume and spares the surrounding normal tissue. It is achieved through modulation of the intensity of photon beams, via the simultaneous variation of three parameters during the treatment delivery: gantry rotation speed; treatment aperture, shaped by multileaf collimator (MLC) leaves; and dose rate.

Multileaf collimators are used to shield anatomic structures from photon radiation and to modulate the field of incident photon fluence.^3^ Due to the MLC’s physical design, it has a great impact on the photon radiation field. Taking the Varian (Varian Associates, Palo Alto, CA, USA) linear accelerator (LINAC) as an example, the MLC works with the primary collimator or jaw (the second) are fixed or follow the open window of the MLC dynamically.^4^ Leakage and transmission through the MLC leaf directly affect plan quality in relation to dosimetry. Multileaf collimator leaf transmission was evaluated in terms of plan quality in Ref. [5]. Furthermore, highly modulated dose distribution requires that the MLCs move at high speed when the gantry rotates. It is worthwhile to consider the relationship between plan dosimetric variation (or plan quality) and maximum MLC leaf speed change. Rapid MLC speed could possibly improve plan quality. However, it could go too far, for the following three reasons. First, the fast leaf motion during gantry rotation may be affected by interleaf friction or MLC motor problems that result in leaf position errors. As demonstrated by Kerns et al.^6^ and Park et al.,^7^ both MLC speed and MLC acceleration exert a considerable effect on VMAT delivery accuracy. Based on clinical uses, it is not reasonable that maximum leaf speed be as rapid as possible. Second, both the dose rate and gantry speed in VMAT are allowed to vary, in addition to MLC leaf positions, to generate a highly conformal target dose distribution with minimal delivery time and monitor units (MUs). It is worthwhile comprehensively investigating the influence of maximum MLC leaf speed on the plan quality of VMAT. Third, plan quality is the best metric for determining the optimal maximum leaf speed for VMAT in clinical practice. It is widely believed that the plan quality may be improved by increasing the MLC leaf speed. However, there has been no thorough study about the benefit of the maximum MLC leaf speed parameter in the LINAC. Validating the optimal maximum MLC leaf speed parameter for clinical implementation is of great interest in clinical practice.

The main objective of this research was to determine the influence of the maximum MLC leaf speed (MMLS) on plan quality. The range of MMLSs was 1 ~ 10 cm/s to cover all MMLS available in the LINAC market. The upper limit of MMLSs extent is 10 cm/s, far beyond the maximum values of MMLS in commercial LINAC to make sure this study reached MMLS large enough. In order to avoid the subjective bias from different planners,^8^ all plans were optimized with an automatic plan tool. Locally advanced nasopharyngeal carcinoma (NPC) and rectal cancer VMAT plans are presented as complex and simple clinical circumstances. Plans for both conditions were evaluated to determine the plan quality dependence of MMLS and the optimal MMLS.

## 2 MATERIALS AND METHODS

### 2.A MLC leaf speed simulation

Seven MLCs with different maximum leaf speeds (1.0, 1.5, 2.25, 3.5, 5.0, 7.5, and 10.0 cm/s) were configured with the Varian Novalis TX LINAC (Varian Medical Systems, Inc., Palo Alto, CA, USA)^9^ in the Pinnacle^3^ treatment‐planning system (TPS, Version 9.10, Philips Radiation Oncology Systems, Fitchburg, WI, USA). Hereafter the MLCs are referred to as S1, S1.5, S2.25, S3.5, S5, S7.5, and S10, respectively. The S2.25, S3.5, and S5 MLC models represent MLC leaf speed parameters currently available in the market, such as the single‐layer high‐definition 120‐leaf MLC (Varian Medical Systems, Inc.), 160‐leaf Agility MLC (Elekta, AB, Stockholm, Sweden)^10^ and Halcyon^TM^ dual‐layer MLC system(Varian Medical Systems, Inc.).^11^ The seven LINACs are all equipped with high‐definition multileaf collimators (HD‐120 HD‐MLC), with leaf widths of 5 mm for the central 40 leaf pairs and 10 mm for the outer 20 leaf pairs.

### 2.B Patient information and target volume definition

Six patients diagnosed with NPC were selected, representing complex clinic cases. All patient characteristics, including cancer stage, disease site, and the volume of targets are listed in the top part of Table 1. All NPC patients with biopsy‐proven, newly diagnosed, nonmetastatic NPC were treated with VMAT at our center. Patients were staged according to the 2010 American Joint Committee on Cancer staging system.^12^ The head, neck, and shoulders were immobilized using a perforated, thermoplastic head, and neck mask in a supine position. Treatment‐planning CT images extending from the vertex of the skull to the carina of trachea were obtained and indexed every 3 mm. The target volumes were delineated using an institutional treatment protocol, which is defined in Ref. [13]. Gross tumor volume (GTV) included the periphery of the GTV of the primary tumor (GTVnx), metastatic retropharyngeal lymph nodes (GTVrpn), and nodal gross tumor volume (GTVnd), as determined by imaging, and clinical and endoscopic findings. Two clinical target volumes (CTVs) were defined: CTV1 and CTV2. CTV1 was defined as the high‐risk region, which included GTVnx plus a 5‐ to 10‐mm margin; CTV1 also encompassed the entire nasopharynx, retropharyngeal lymph node regions, parapharyngeal space, and any high‐risk nodal regions. CTV2 was defined as the low‐risk lymph node regions. The planning target volume (PTV) included allowances for setup error and organ movement during the treatment process and was generally extended by 3 mm on the basis of the GTVs and CTVs. PTV1 of CTV1 (i.e., the high‐risk regions), and PTV2 of CTV2 (i.e., low‐risk regions) were derived using the simultaneous integrated boost technique. Briefly, the prescribed doses were 73.92, 73.92, 69.96, and 60.06 Gy, delivered within 33 fractions to PGTVnx, GTVrpn, GTVnd, and PTV1, respectively. The total dose of PTV2 was given in 28 fractions at 1.82 Gy per daily fraction in clinic. For simplicity and to plan dosimetric comparisons in our study, PTV1 and PTV2 were added up as PTV1, given as 60.06 Gy in 33 fractions.

**Table 1:** General information and disease characteristics from six patients with NPC and nine patients with rectal carcinoma.

Disease sites: NPC		Target volume (cm^3^)			
ID	Stage	PGTV	GTVnd	GTVrpn	PTV1
1	III	133.4	5.7	3.4	781.1
2	III	71.7	1.5	2.9	597.1
3	IVA	90.1	20.6	0.6	593.8
4	III	64.2	10.7	1.9	899.0
5	III	50.7	26.6	11.2	790.0
6	III	58.7	46.5	5.0	876.4

Disease sites: rectum		Target volume (cm^3^)
ID	Stage	PTV
1	IIIB	1517.3
2	IIIA	1666.9
3	IIIB	1391.8
4	IIIB	1518.2
5	IIIC	1039.8
6	IIIB	1532.0
7	IIA	1533.8
8	IIIB	1728.2
9	IIIC	1609.3

Nine patients diagnosed with rectal cancer were selected, representing a simple scenario. Radiotherapy was performed with a prescription dose of 50 in 2 Gy per fraction. The radiation oncologists delineated the target volume and organs at risk (OARs) on a planning CT scan. The planning CT and treatment of the patients were performed with prone positioning, with full bladder. The CTV included the primary tumor, and the mesorectal, presacral and internal iliac lymph nodes. CTV was enlarged in all directions by 10 mm to define the PTV. General information and disease characteristics from the six rectal patients are listed in the bottom part of Table 1.

The planning organ‐at‐risk volume (PRV) was created for the spinal cord by adding a 5‐mm margin and was denoted as spinal cord PRV. A supporting structure for the normal tissue (NT) was created for an area surrounding the PTV range and evaluated the general low‐dose loading in the scanned area. NT represents the external contour of the patient’s body minus the PTV with an additional distance of 0.5 cm.^5^

### 2.C Planning method

Seven plans were designed for each patient, with seven LINACs configured with different MMLSs. Each plan was designed with the same optimization parameter by the mdaccAutoPlan system and evaluated quantitatively for each patient. Plans can be generated by one button click in maccAutoPlan system which is as a plug‐in to the Pinnacle^3^ TPS.^8^ The VMAT algorithm in Pinnacle3 TPS is SmartArc and the five factors (MMLS, minimum rotation speed of gantry, target size, maximum dose rate, and maximum dose gradient) are contributed the plan quality in the stage of segmentation of fluence map. A description of the SmartArc optimization algorithm was published by Bzdusek et al.^14^ The mdaccAutoPlan system was developed based on our clinical protocol, with authorization from developer Zhang’s team. The quality of the planning outcome depends on the method followed by each planner,^15^ so the use of automated planning decreased interoperator variability^16^ and guarantee high‐quality VMAT and IMRT treatment plans in our study^17^. In our study, VMAT plans were calculated using 6‐MV photons, with a maximum variable dose rate of 600 MU/min. Double arcs with coplanar arcs of 360° shared the same isocenter, using opposite rotation (clockwise and counter clockwise). The collimator was always rotated to 10° and 350°, respectively, in two arcs, to avoid a tongue and groove effect. The maximum rotation time of each arc was set to 60 s, to guarantee the leaf travel was as rapid as possible. The gantry angle spacing was 4°. The calculation voxel size was isotropic and 4 mm.

### 2.D Plan evaluation and statistical analyses

Plan quality was evaluated by plan scores which was introduced by QUASI‐MOD group.^18^ Plan scores implemented by the Plan Quality Algorithm (PQA) tool that is composed of the Plan Quality Metric (PQM) components in PlanIQ^TM^ software (Sun Nuclear, Melbourne, FL, USA). The PQA was employed here as an objective method to quantify a plan's quality, particularly in terms of meeting clear and specific treatment‐plan goals. PQM removes any ambiguity from the plan objectives and provides a fair comparison of plan results.^16^

There were 40 and 21 components of the PQM for the NPC and rectal cases, respectively. Each plan was given a score based on a unique PQA, of which the PQM value function used to calculate a point value was based on the submetric. Descriptions of each PQM of the NPC and rectal cases are shown in Tables 2 and 3, respectively. Full scores for the rectal plan and NPC plan were 36 and 102, respectively. PTV and OAR dose metrics were evaluated for all plans. The plans reflected all optimization objectives routinely employed in our clinic,^19^ which are more stringent than similar objectives from RTOG protocols.^20^ The Homogeneity Index (HI) was defined as in Ref. [21]. Conformation Number (CN) represents the dose fit of the PTV, relative to the volume covered by the prescribed isodose lines, which are defined in Ref. [22]. V_n Gy_ (%) is the percentage of the organ volume receiving ≥ n Gy.^5^ D_v cc_ and Dmean are the near‐maximum absorbed dose (where V is a small fractional volume) and average absorbed doses delivered to each OAR, respectively.^23^

**Table 2:** List of metrics, definition, and PQM value range used to form the PQM algorithms for NPC patient plans.

Plan quality metric component	Objective(s)	Score	
[ROI] metrics	Endpoint [optimal]	min	max
[BRAIN STEM PRV] V[60.0 Gy] (cc)	<10 [≤0.01]	0	2
[BRAIN STEM] V[54.0 Gy] (cc)	<10 [≤0.01]	0	4
[GTVND] homogeneity index^#^ [69.96 Gy]	<1 [≤0]	0	3
[GTVND] V[69.96 Gy] (%)	>94.5 [≥95]	0	5
[GTVND] V[74.86 Gy] (cc)	<10 [≤1]	0	2
[GTVND+(PGTVNX + GTVRPN+0.3)] conformation number [69.96 Gy]	>0.25 [≥0.85]	0	2
[GTVRPN] homogeneity index [73.92 Gy]	<1 [≤0]	0	3
[GTVRPN] V[73.92 Gy] (%)	>94.5 [≥95]	0	5
[GTVRPN] V[79.09 Gy] (cc)	<10 [≤1]	0	3
[LARYNX] V[40.0 Gy] (%)	<60 [≤30]	0	2
[LENS L] V[9.0 Gy] (cc)	<0.1 [≤0.01]	0	3
[LENS R] V[9.0 Gy] (cc)	<0.1 [≤0.01]	0	3
[MANDIBLE L] V[60.0 Gy] (%)	<20 [≤5]	0	2
[MANDIBLE R] V[60.0 Gy] (%)	<20 [≤5]	0	2
[NT] D[0.01 cc] (Gy)	<66.07 [≤57.06]	0	1
[NT] V[20.0 Gy] (%)	<90 [≤50]	0	1
[NT] V[30.0 Gy] (%)	<80 [≤20]	0	1
[OPTIC CHIASM] V[54.0 Gy] (%)	<1 [≤0]	0	2
[OPTIC NERVE L] V[54.0 Gy] (%)	<10 [≤0.1]	0	2
[OPTIC NERVE R] V[54.0 Gy] (%)	<10 [≤0.1]	0	2
[PAROTID L] V[20.0 Gy] (%)	<90 [≤60]	0	1
[PAROTID L] V[30.0 Gy] (%)	<65 [≤45]	0	2
[PAROTID R] V[20.0 Gy] (%)	<90 [≤60]	0	1
[PAROTID R] V[30.0 Gy] (%)	<65 [≤45]	0	2
[PGTVNX] homogeneity index [73.92 Gy]	<1 [≤0]	0	3
[PGTVNX] V[73.92 Gy] (%)	>94.5 [≥95]	0	5
[PGTVNX + GTVRPN] conformation number [73.92 Gy]	>0.25 [≥0.85]	0	4
[PGTVNX + GTVRPN] V[79.09 Gy] (%)	<50 [≤10]	0	3
[PTV1‐(PGTVNX + GTVRPN+GTVND)] homogeneity index [60.06 Gy]	<1 [≤0]	0	3
[PTV1‐(PGTVNX + GTVRPN+GTVND)] V[64.26 Gy] (%)	<80 [≤10]	0	3
[PTV1] conformation number [60.06 Gy]	>0.65 [≥0.87]	0	2
[PTV1] V[60.06 Gy] (%)	>94.5 [≥95]	0	5
[SPINAL CORD PRV] V[45.0 Gy] (cc)	<0.1 [≤0]	0	2
[SPINAL CORD] V[40.0 Gy] (cc)	<0.1 [≤0]	0	4
[TEMPORAL LOBE L] V[54.0 Gy] (%)	<5 [≤1]	0	2
[TEMPORAL LOBE R] V[54.0 Gy] (%)	<5 [≤1]	0	2
[THYROID GLAND] V[40.0 Gy] (%)	<70 [≤40]	0	2
[TMJ L] V[50.0 Gy] (%)	<40 [≤1]	0	2
[TMJ R] V[50.0 Gy] (%)	<40 [≤1]	0	2
[TRACHEA] V[40.0 Gy] (%)	<70 [≤10]	0	2
Total [40 metrics]		0	102

The first column displays the PQM components, the structure presents in square brackets with the corresponding metric. The second column shows the endpoint and optimal structure metrics that are corresponds to the minimum and maximum score, respectively, in the third column.

**Table 3:** List of metrics, definition, and PQM value range used to form the PQM algorithm for rectal cancer patient plan

Plan quality metric component	Objective(s)	Score	
[ROI] metric	Endpoint [optimal]	min	max
[PTV] V[53.5 Gy] (%)	<10 [≤0.1]	0	3
[PTV] V[50.0 Gy] (%)	>94.5 [≥95]	0	4
[PTV] homogeneity index [50.0 Gy]	<1 [≤0]	0	3
[PTV] conformation number [50.0 Gy]	>0.65 [≥0.87]	0	3
[NT] V[10.0 Gy] (%)	<99 [≤70]	0	1
[NT] V[30.0 Gy] (%)	<50 [≤10]	0	1
[NT] V[20.0 Gy] (%)	<95 [≤50]	0	1
[NT] D[0.01 cc] (Gy)	<55 [≤47.5]	0	1
[INTESTINE] V[52.0 Gy] (cc)	<10 [≤0.01]	0	1
[INTESTINE] V[40.0 Gy] (%)	<20 [≤1]	0	2
[INTESTINE] V[30.0 Gy] (%)	<30 [≤5]	0	2
[FEMUR R] V[30.0 Gy] (%)	<50 [≤10]	0	1
[FEMUR R] mean dose (Gy)	<20 [≤12]	0	1
[FEMUR L] V[30.0 Gy] (%)	<50 [≤10]	0	1
[FEMUR L] mean dose (Gy)	<20 [≤12]	0	1
[COLON] V[52.0 Gy] (cc)	<20 [≤0.01]	0	1
[COLON] V[40.0 Gy] (%)	<30 [≤5]	0	2
[COLON] V[30.0 Gy] (%)	<60 [≤30]	0	2
[BLADDER] V[52.0 Gy] (cc)	<60 [≤1]	0	1
[BLADDER] V[40.0 Gy] (%)	<80 [≤40]	0	2
[BLADDER] V[30.0 Gy] (%)	<95 [≤60]	0	2
Total [21 metrics]		0	36

The first column displays the plan quality metric components, the structure presents in square brackets with the corresponding metric. The second column shows the endpoint and optimal structure metrics that are corresponds to the minimum and maximum score, respectively, in the third column.

In mathematics, a “stationary point” is an input to a differentiable function such that the derivative is zero. In order to determine the optimal MMLS, the stationary point of maximum MLC speed (MMSSP) was defined as the relative change of plan score that is first <5%, as MMLS increases. MMSSP is close to 0, but could not equal to 0, because the MMLS was not a continuous variable in our study.

Statistical analyses of plan scores and each PQM components values from seven MMLS groups for each disease sites were performed with the SPSS Program, version 23.0 (Statistical Package for the Social Sciences; SPSS, Chicago, IL, USA). The Shapiro–Wilk test was applied to verify if the data were normally distributed. Data were submitted to one‐way ANOVA (analysis of variance). Bonferroni’s post‐hoc multiple comparison test was used for pairwise comparisons. In the case of non‐normally distributed values or variance heterogeneity, the nonparametric Kruskal–Wallis test was adopted for comparing seven groups of data. A significance level of 0.05 was used for all tests.

## 3 RESULTS AND DISCUSSION

**Fig. 1:**
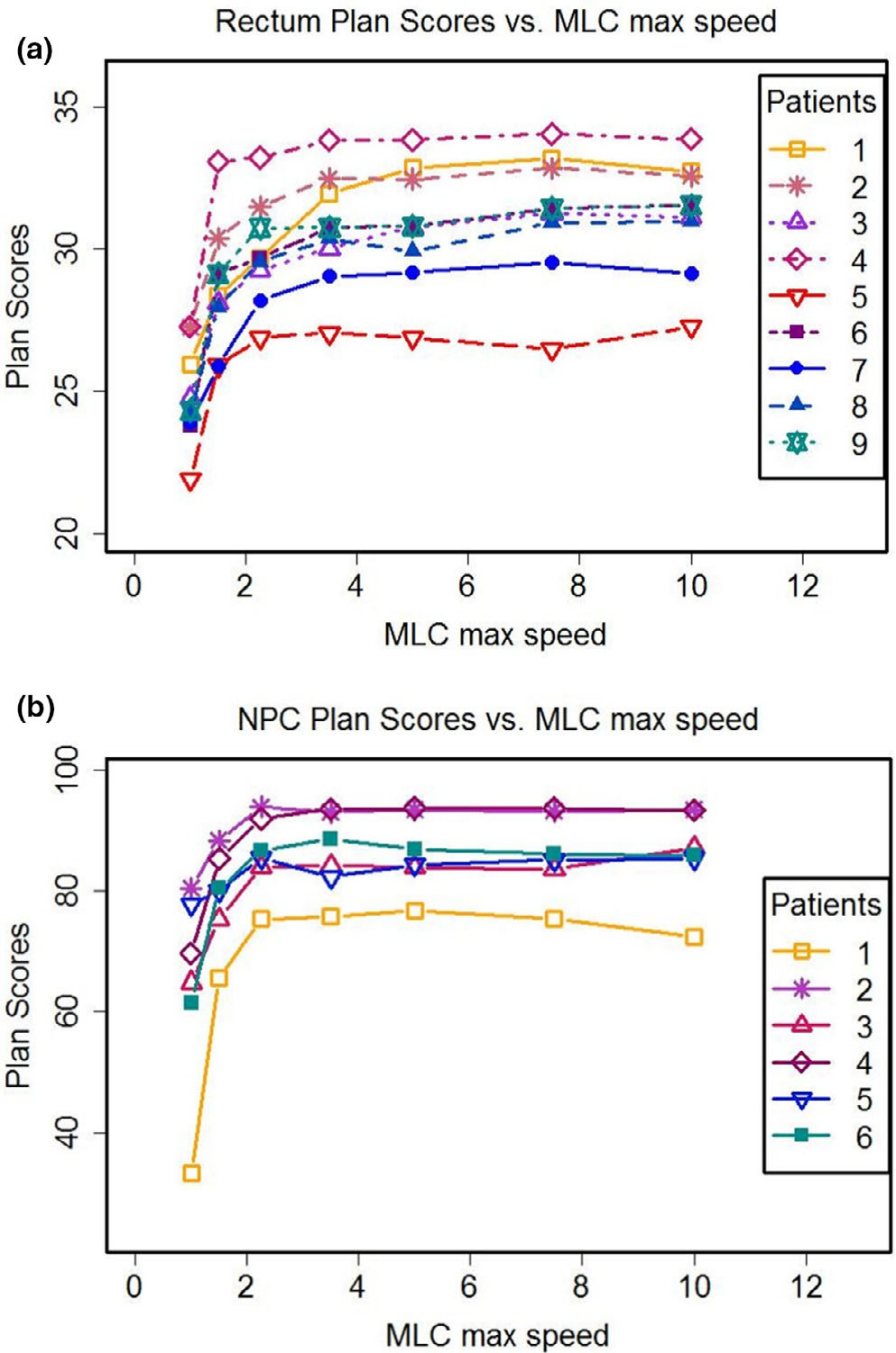
Plan scores versus maximum MLC leaf speed in nine patients with rectal cancer (a) and six patients with locally advanced nasopharyngeal carcinoma (NPC) (b).

VMAT treatment plans were generated for each patient in this study, with the seven MLCs applied. In total, 63 rectal plans and 42 NPC plans were generated and analyzed for these patients. Two well‐defined PQMs of rectal and NPC cases were used for qualitative and quantitative analysis of plan quality for 105 plans. Figure 1 shows a summary of the PQM scores of the rectal cases and NPC cases, based on seven configured LINACs with the various MMLSs. For the rectal cancer cases, plan scores dramatically increased when MMLS increased from 1 to 3.5 cm/s. Plan scores increased slowly when MMLS increased above 3.5 cm/s. The trend indicated that there was better plan quality with higher MMLS in most test cases. This implies that high leaf speeds helped smooth‐out variations in dose distributions, either through small fields or by the leaves blocking high‐dose region for OARs. Plan scores for the NPC case showed the same trends. Due to the larger target volume in NPC cases, it is more difficult to achieve plan design goal, and to maintain the conformal and homogenous dose to the target. However, MMLS limitation was present in about equal proportions between the two sites for plan quality.

**Fig. 2:**
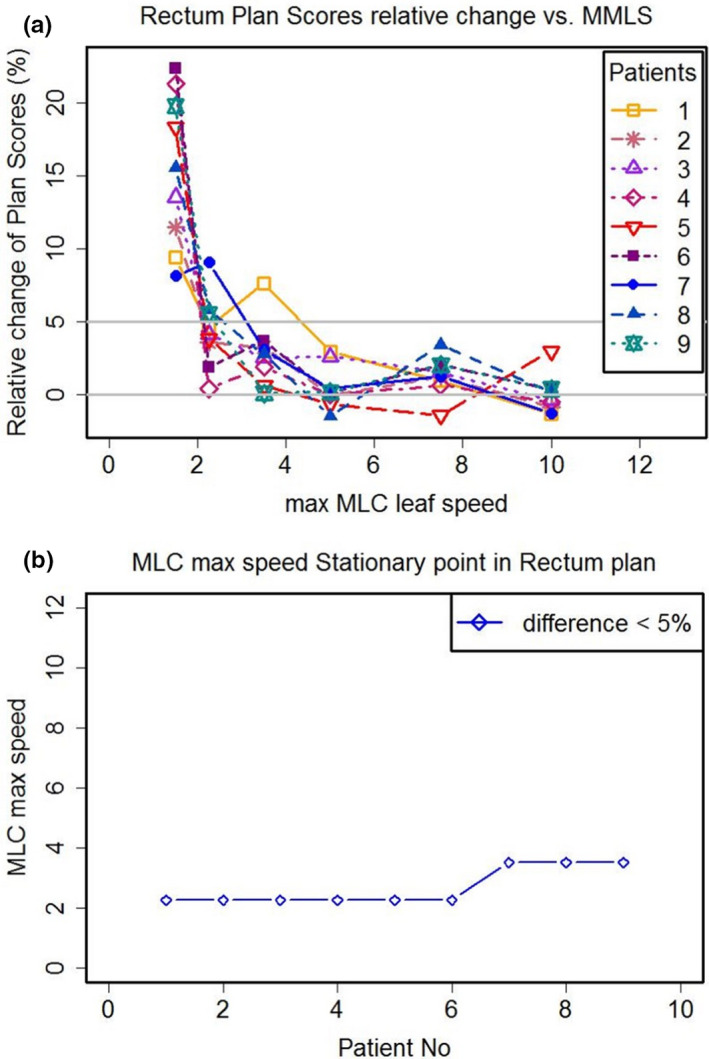
(a) The relative change of plan scores for each patient with rectal cancer, from Fig 1 left panel. (b) The stationary point of maximum multi‐leaf collimator speed (MMSSP) for each patient under the relative change of plan scores < 5%.

Detailed plan quality variations in the seven types of MMLS plans are shown in Figure 2(a) for rectal cancer cases. The relative change of plan quality scores with all rectal cancer cases decreased at lower leaf speed when MMLS was below 2.25 cm/s. After that, the relative change of plan scores was mostly within 5%. This scenario was conspicuous in rectal cases as well, even more apparent. When MMLS was above 3.5 cm/s, an optimum plan with little further upgrade in plan quality was produced for small increases in leaf speed. It is more possibly that the MMLS of 3.5cm/s met the requirement to deliver the plan. MMSSP is the specific MMLS at which the relative change of plan scores first drops in a tiny interval (<5%). Figure 2(b) plots the MMSSPs when the relative change of plan scores was within 5%. For the rectal plans, MMSSPs were 2.25 for six patients (66.67% of total patients) and 3.5 for the other three patients.

**Fig. 3:**
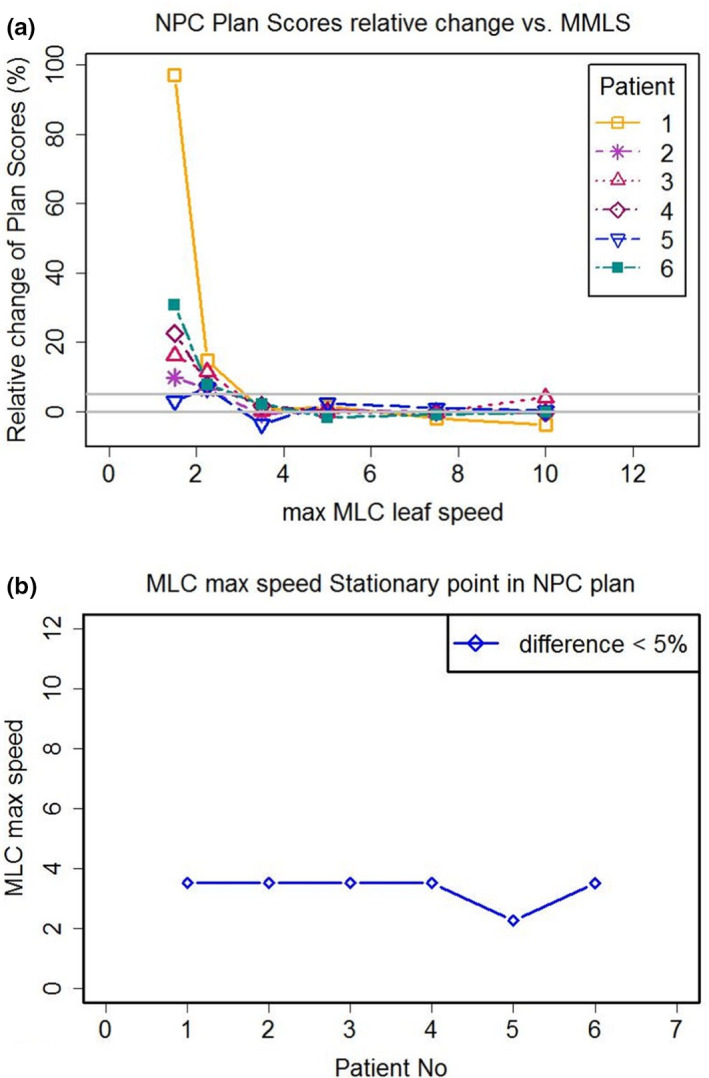
(a) The relative changes of plan scores for each patient with locally advanced nasopharyngeal carcinoma (NPC) from Fig 1 right panel. (b) The stationary point of maximum multi‐leaf collimator speed (MMSSP) for each patient under the relative change of plan scores < 5% (blue line).

Figure 3 displays the relative change of plan scores for the NPC cases and the MMSSP values based on the relative change of plan scores. Generally, the relative changes of plan scores for NPC cases showed a more obvious trend than did the rectal cases. For NPC plans, the relative change of plan quality declined with increasing MMLS when MMLS was <3.5 cm/s. The relative change of plan scores decreased slightly when MMLS was larger than 3.5 cm/s. For NPC plans, MMSSP was 3.5 for five patients (83.33% of total patients) and 2.25 for the other patient.

For plan scores of rectal cancer cases, the pairwise ANOVAs showed (a) the plan scores of S1 plan did not differ significantly from that of S1.5 plan, (b) while S1 plan was significantly difference to S2.25, S3.5, S5, S7.5, and S10 plans methods (*P* < 0.01). (c) Except S1 plan, the five MMLS plans did not differ significantly from each other. For NPC cases, the pairwise ANOVAs showed (a) S1 plan was significantly difference to other five MMLS plans methods (*P* < 0.01), (b) the five MMLS plans did not differ significantly from each other. The statistical results of plan scores from seven MMLS type plans in both rectal cases and NPC cases were consistent with MMSSP result in both Figs. 2 and 3.

The corresponding analysis with PQM components in two disease sites yielded similar results, but less pairs with significant differences. For PQM components of rectal cancer cases, the 5 of 21 components which show the statistically differences are reported in Table 4, while the other MMLS type plans did not differ significantly from each other. In particular, the CN of PTV in S1 plan for rectal cases showed significant difference with other six MMLS type plans, respectively. The CN value of PTV in S1.5 plan from rectal cases was significantly lower than that in S7.5 plans (*P* = 0.044). For PQM components of NPC cancer cases, the 9 of 40 components which show the statistically differences are reported in Table 5, while the other MMLS type plans did not differ significantly from each other. The comparison in terms of CN of [GTVnd + (PGTVnx + GTVrpn + 0.3)] with prescription dose 69.96 Gy in S1 plan from NPC cases was statistically significant with other six type plans (*P* < 0.05). A similar behavior was founded for V_40Gy_ of larynx (*P* = 0) in NPC cases.

**Table 4:** Results in terms of *P*‐values < 0.05 for Bonferroni’s post‐hoc multiple comparison test or nonparametric Kruskal–Wallis test obtained for PQM components.

PQM	*P*‐values of pair comparison																				
[ROI] metrics	S1 vs S1.5	S1 vsS2.25	S1 vs S3.5	S1 vs S5	S1 vs S7.5	S1 vs S10	S1.5 vs S2.25	S1.5 vs S3.5	S1.5vs S5	S1.5 vs S7.5	S1.5 vs S10	S2.25 vs S3.5	S2.25 vs S5	S2.25 vs S7.5	S2.25 vs S10	S3.5 vs S5	S3.5 vs S7.5	S3.5 vs S10	S5 vs s7.5	S5 vs S10	S7.5 vs S10
[PTV] V[53.5 Gy] (%)	–	–	0.004	0.001	0.000	0.000	–	–	–	0.025	0.034	–	–	–	–	–	–	–	–	–	–
[PTV] homogeneity index [50.0 Gy]	–	–	0.005	0.004	0.000	0.000	–	–	–	–	–	–	–	–	–	–	–	–	–	–	–
[PTV] conformation number [50.0 Gy]	0.004	0.000	0.000	0.000	0.000	0.000	–	–	–	0.044	–	–	–	–	‐	–	–	–	–	–	–
[NT] D[0.01 cc] (Gy)	–	–	0.003	0.001	0.004	0.011	–	–	–	–	–	–	–	–	–	–	–	–	–	–	–
[BLADDER] V[52.0 Gy] (cc)	–	–	–	0.036	0.003	0.006	–	–	–	–	–	–	–	–	–	–	–	–	–	–	–
Total plan scores [21 Metrics]	–	0.005	0.005	0.004	0.005	0.005	–	–	–	–	–	–	–	–	–	–	–	–	–	–	–

Comparisons were performed among seven MMLS type plans in rectal cancer cases.

**Table 5:** Results in terms of *P*‐values < 0.05 for Bonferroni’s post‐hoc multiple comparison test or the nonparametric Kruskal–Wallis test obtained for PQM components.

PQM	*P*‐values of pair comparison																				
[ROI] metrics	S1 vs S1.5	S1 vs S2.25	S1 vs S3.5	S1 vs S5	S1 vs S7.5	S1 vs S10	S1.5 vs S2.25	S1.5 vs S3.5	S1.5vs S5	S1.5 vs S7.5	S1.5 vs S10	S2.25 vs S3.5	S2.25 vs S5	S2.25 vs S7.5	S2.25 vs S10	S3.5 vs S5	S3.5 vs S7.5	S3.5 vs S10	S5 vs s7.5	S5 vs S10	S7.5 vs S10
[GTVND] homogeneity index^#^ [69.96 Gy]	–	0.038	–	0.045	0.045	–	–	–	–	–	–	–	–	–	–	–	–	–	–	–	–
[GTVND] V[74.86 Gy] (cc)	–	–	0.031	0.005	0.018	0.020	–	–	–	–	–	–	–	–	–	–	–	–	–	–	–
[GTVND+(PGTVNX + GTVRPN+0.3)] Conformation Number [69.96 Gy]	0.012	0.000	0.000	0.000	0.000	0.000	–	–	–	–	–	–	–	–	–	–	–	–	–	–	–
[GTVRPN] V[79.09 Gy] (cc)	–	–	–	0.014	0.025	–	–	–	–	–	–	–	–	–	–	–	–	–	–	–	–
[LARYNX] V[40.0 Gy] (%)	0.000	0.000	0.000	0.000	0.000	0.000	–	–	–	–	–	–	–	–	–	–	–	–	–	–	–
[NT] D[0.01 cc] (Gy)	–	–	–	–	0.014	0.033	–	–	–	–	–	–	–	–	–	–	–	–	–	–	–
[PGTVNX + GTVRPN] V[79.09 Gy] (%)	–	–	–	0.026	–	–	–	–	–	–	–	–	–	–	–	–	–	–	–	–	–
[PTV1‐(PGTVNX + GTVRPN+GTVND)] V[64.26Gy] (%)	–	0.001	0.000	0.000	0.000	0.000	–	–	0.049	–	–	–	–	–	–	–	–	–	–	–	–
[PTV1] conformation number [60.06 Gy]	–	–	0.035	0.038	0.045	0.010	–	–	–	–	–	–	–	–	–	–	–	–	–	–	–
Total plan scores y	–	0.005	0.005	0.004	0.005	0.005	–	–	–	–	–	–	–	–	–	–	–	–	–	–	–

Comparisons were performed among seven MMLS type plans of NPC cancer cases.

**Fig. 4:**
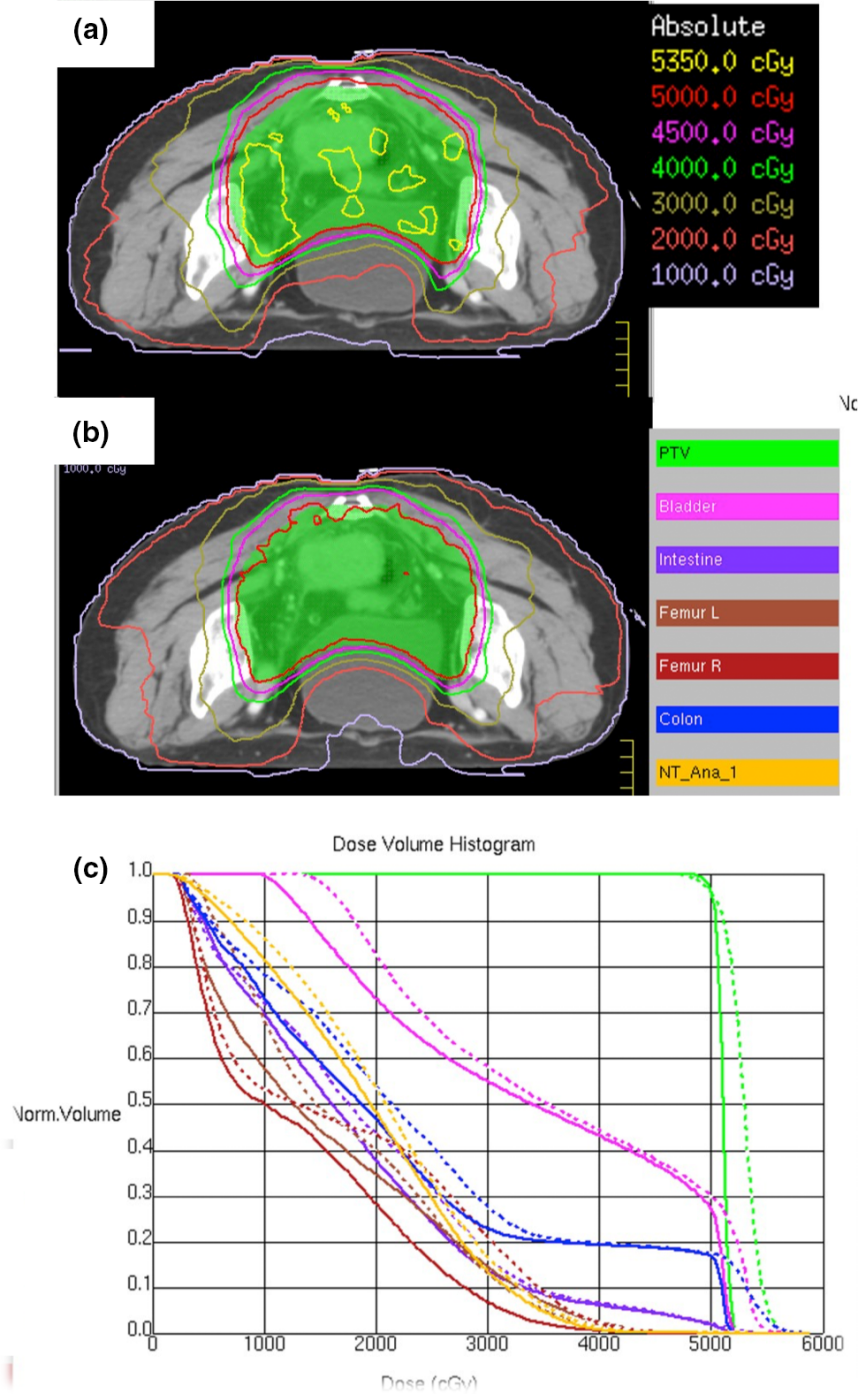
Comparison between S1 and S3.5 plans for patient #6 with rectal cancer. Axial dose distribution of S1 plan (a) and S3.5 plan (b) and corresponding dose volume histograms (DVHs) (c) for Patient #6. The dose distributions show planning target volume (PTV) prescribed to 5000 cGy (red line). The DVHs of the S1 plan (solid lines) and S3.5 plan (dashed lines) include the following ROIs: bladder (purple), intestine (slate‐blue), colon (blue), left femur head (brown), right femur head (maroon), normal tissue (orange) and PTV (green). The S1 plan for Patient #6 achieved fewer dose evaluation criteria than did the S3.5 plan. This is shown by the higher maximum dose for the bladder, in which the S1 plan exceeded the criteria limit.

Representative VMAT plans for a rectal cancer patient, with axial dose distribution and the DVHs of PTV and OARs from S1 and S3.5 plans, are shown in Fig. 4. As shown in the top‐left and bottom‐left panels of Fig. 4, the S3.5 plan not only achieved better conformity to the 95% isodose line (50 Gy) of the PTV, but also included fewer hot spots — the 107% isodose line (53.5 Gy) — than the S1 plan did. In addition, the S3.5 plan produced steeper DVHs than the S1 plan did in the high‐dose ranges, as shown in Fig. 4(c). That means that the irradiation dose curves were more constricted around the target in the S1 plan. For selected OARs, the normalized bladder volume in the S3.5 plan was smaller than in the S1 plan, in the 10 to 35 Gy dose range, and even over 50 Gy. The percentage volume of the colon was significantly smaller throughout the entire dose range in the S3.5 plan than in the S1 plan, especially between 10 and 30 Gy, and over 50 Gy. Interestingly, the volume of left and right femur heads received radiation doses below 40 Gy in the S3.5 plan were smaller than in the S1 plan. The same numeric trend was observed in the NT DVH.

**Fig. 5:**
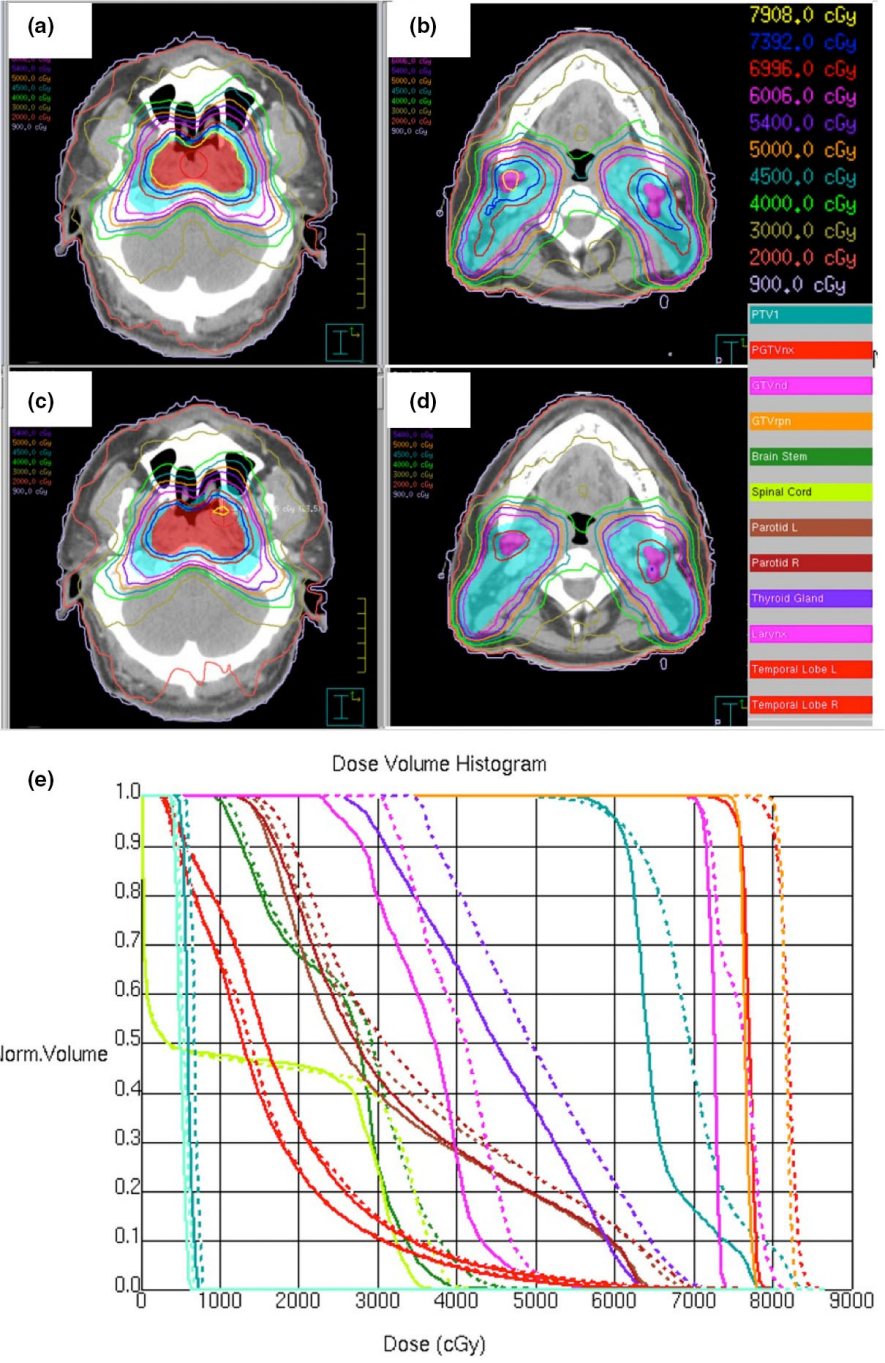
Comparison between S1 plan and S3.5 plans for a patient with locally advanced nasopharyngeal carcinoma. Axial dose distribution for S1 plan (a, b) and S3.5 plan (c, d), with corresponding DVHs (e) for Patient #4. The dose distributions show PGTVnx and GTVrpn prescribed to 73.92 Gy (red line), GTVnd prescribed to 69.96 Gy (purple), and PTV1 prescribed to 60.06 Gy (teal line). The DVHs for the S1 plan (solid lines) and S3.5 plan (dashed lines) include the following ROIs: brainstem (forest green), spinal cord (yellow‐green), larynx (purple), left parotid (brown), right parotid (maroon), left Lens (sky blue), right lens (steel‐blue), and left and right temporal lobes (tomato red). Patient #4, in whom the target exceeded more dose evaluation criteria than clinically required, and lots of hot spots are on target. This is shown by the higher intermediate dose for the parotids, in which the S1 plan exceeded the criteria limit.

Figure 5 shows one NPC case with the three targets prescribed to receive 73.92 Gy (PGTVnx, GTVrpn), 69.96 Gy (GTVnd), and 60.06 Gy (PTV1) in 33 fractions. The critical structures were the spinal cord, brain stem, larynx, left and right parotids, left and right lenses, and left and right temporal lobes. Two‐dimensional dose distributions on the transverse plane of S1 and S3.5 plans are shown in the top‐ and bottom‐left panels of Fig. 5. The second to fifth isodose lines plot 95% of each prescribed dose. A DVH comparison of S1 (dashed lines) and S3.5 (solid lines) plans is shown in the right panel of Fig. 5. Similar to the results observed in the rectal cancer patients, better plan quality was achieved with the S3.5 plan in NPC patients.

Chen et al.^18^ investigated how leaf motion constraints affect the quality, accuracy, and efficiency of IMAT plans. Using fluence imported into an in‐house sequencer designed to generate IMAT plans, they investigated the effect of applying a restriction on the MLC leaf motion, in terms of the distance that the MLC leaves could travel per degree over a range of 1 to 30 mm/°. They found that there was a significant impact on the quality, efficiency, and accuracy of the plans delivered, especially in complex treatments. They recommended a leaf motion constraint of 2 to 3 mm/° and noted that, as the motion constraint was relaxed, the delivery times increased. Their studies were based on IMAT, whereas VMAT is an improved technique. VMAT is widely accepted and used in clinical practice. The dependence of VMAT plans on the maximum allowed MLC speed was investigated in this study. A survey of how one machine parameter (maximum leaf motion) affected VMAT plan quality for both simple and complex plans was carried out. Our results showed that as MMLS increased, so did VMAT plan quality, resulting in better conformity, more homogenous dose distributions and higher plan quality scores. For simple clinical scenarios, such as the rectal cases, MMSSPs were 2.25 cm/s for six patients and 3.5 cm/s for three patients when the relative change of plan scores was less than 5%. For complex clinical scenarios, such as NPC, MMSSPs were 3.5 cm/s for one patient and 2.25 ~ 3.5 cm/s for the other five. In our study, the target size varies from 1039.8 to 1728.2 cm^3^ in rectum cases, from 50.7 to 133.4 cm^3^ (PGTV) and from 597.1 to 899.0 cm^3^ (PTV1) in NPC cases, as shown in Table 1. Although the tumor (target) size varies from the patient to patients in both NPC and rectum case, the quality of VMAT plans presented the same trend with MMLS changed: the plan quality is greatly improved as MMLS increases from 1 to 3.5 cm/s; above that, the quality change is marginal. It demonstrated that the MMLS have influences on plan quality regardless of the tumor size. A limitation of this study was that the impact on the dosimetric effect from the interactions of dose rate, gantry speed, and MLC speed were not considered. In this study, we focused only on the dosimetric effect from the maximum MLC leaf speed. Two major characteristics of MLC have been exclusively studied in the dosimetric effect of plan quality: the MLC leaf speed here and MLC transmission in Ref [5]. Our future study could incorporate the interactions of dose rate, gantry speed, and MLC speed, to obtain an even more abundant representation of the dosimetric effect.

## 4 CONCLUSIONS

This work indicates that the maximum leaf speed of MLCs has an influence on the quality of VMAT plans in NPC cases and rectal cancer cases. The quality of VMAT plans is greatly improved as MMLS increases from 1 to 3.5 cm/s; above that, the quality change is marginal.

## AUTHORS CONTRIBUTION

All authors discussed and conceived of the study design. Jiayun Chen performed case selection and data analysis, score metric development, and drafted the manuscript. Weijie Cui did the initial validation of the project. Qi Fu and Haojia Zhang did the treatment planning of the cases. Xiaodong Huang contoured the targets and organs at risk of the case. Hanfei and Wenlong Xia evaluated all plans and provided meaningful suggestions about the plan evaluation. Bin Liang participated in discussions about the data analysis and provided meaningful suggestions. Jianrong Dai guided the study preparation of the manuscript. All authors read, discussed, and approved the final manuscript.

## CONFLICT OF THE INTEREST

None declared.
